# Supplementary material for: Resolving the full spectrum of human genome variation using Linked-Reads
Source: Genome Res. 2019 Apr;29(4):635–45. doi: 10.1101/gr.234443.118 (PMC6442396; doi:10.1101/gr.234443.118)
Supplement: Supplemental Material [file supp_gr.234443.118_Supplemental_longranger-2.2.zip › longranger-2.2.tar.bz2!/lr2.2-public/lib/stan/cmdstan/stan/doc/api/html/classstan_1_1gm_1_1argument__probe.html]

Stan: stan::gm::argument\_probe Class Reference


|  |  |
| --- | --- |
|  | Stan  2.5.0  probability, sampling & optimization |


- Main Page
- Related Pages
- Namespaces
- Classes
- Files

- Class List
- Class Index
- Class Hierarchy
- Class Members

All Classes Namespaces Files Functions Variables Typedefs Enumerations Enumerator Friends Macros Pages

- stan
- gm
- argument\_probe

Public Member Functions |
Protected Attributes |
List of all members

stan::gm::argument\_probe Class Reference

`#include <argument_probe.hpp>`

|  |  |
| --- | --- |
| Public Member Functions | |
|  | argument\_probe (std::vector< argument \* > &valid\_args) |
|  | |
| void | probe\_args (std::stringstream &s) |
|  | |

|  |  |
| --- | --- |
| Protected Attributes | |
| std::vector< argument \* > & | \_arguments |
|  | |

## Detailed Description

Definition at line 14 of file argument\_probe.hpp.

## Constructor & Destructor Documentation

|  |  |  |  |  |  |
| --- | --- | --- | --- | --- | --- |
| stan::gm::argument\_probe::argument\_probe | ( | std::vector< argument \* > & | *valid\_args* | ) |  |

inline

Definition at line 18 of file argument\_probe.hpp.

## Member Function Documentation

|  |  |  |  |  |  |
| --- | --- | --- | --- | --- | --- |
| void stan::gm::argument\_probe::probe\_args | ( | std::stringstream & | *s* | ) |  |

inline

Definition at line 21 of file argument\_probe.hpp.

## Member Data Documentation

std::vector<argument\*>& stan::gm::argument\_probe::\_arguments

protected

Definition at line 31 of file argument\_probe.hpp.

---

The documentation for this class was generated from the following file:

- src/stan/gm/arguments/argument\_probe.hpp


---

[ Stan Home Page ]

*© 2011–2014,
Stan Development Team.*
